# Global Trends in Climate Suitability of Bees: Ups and Downs in a Warming World

**DOI:** 10.3390/insects15020127

**Published:** 2024-02-11

**Authors:** Ehsan Rahimi, Chuleui Jung

**Affiliations:** 1 Agricultural Science and Technology Institute, Andong National University, Andong 36729, Republic of Korea; ehsanrahimi666@gmail.com; 2 Department of Plant Medicals, Andong National University, Andong 36729, Republic of Korea

**Keywords:** species distribution models, pollinators, conservation, climate suitability, ‘*BeeBDC*’ R package

## Abstract

**Simple Summary:**

This study examines the impact of global warming on crucial insect pollinators, particularly bees. Through species distribution models, we evaluated how climate change, specifically under the SSP585 scenario in the year 2070, affects the climate suitability of 1365 bee species worldwide. The results unveiled fluctuating suitability patterns: approximately 65% of bees displayed potential reductions in their distribution ranges due to climate change. Variation was observed across continents, with Africa and Europe experiencing the most pronounced effects and North America being the least affected. Climate change is anticipated to significantly alter bee distributions, potentially disrupting existing plant–bee interactions. This poses ecological challenges that underscore the importance of plant–bee synchrony and the necessity for targeted conservation endeavors.

**Abstract:**

Bees represent vital natural assets contributing significantly to global food production and the maintenance of ecosystems. While studies on climate change effects impacting major pollinators like honeybees and bumblebees raise concerns about global diversity and crop productivity, comprehensive global-scale analyses remain limited. This study explores the repercussions of global warming on 1365 bees across seven families of bees worldwide. To compile a robust global bee occurrence dataset, we utilized the innovative ‘*BeeBDC*’ R package that amalgamated over 18.3 million bee occurrence records sourced from various repositories. Through species distribution models under the SSP585 scenario in the year 2070, we assessed how climate change influences the climate suitability of bees on a global scale, examining the impacts across continents. Our findings suggested that approximately 65% of bees are likely to witness a decrease in their distribution, with reductions averaging between 28% in Australia and 56% in Europe. Moreover, our analysis indicated that climate change’s impact on bees is projected to be more severe in Africa and Europe, while North America is expected to witness a higher number (336) of bees expanding their distribution. Climate change’s anticipated effects on bee distributions could potentially disrupt existing pollinator–plant networks, posing ecological challenges that emphasize the importance of pollinator diversity, synchrony between plants and bees, and the necessity for focused conservation efforts.

## 1. Introduction

The economic value of animal pollination as an ecosystem service in global agriculture ranges from USD 195 billion to USD 387 billion [1]. Additionally, it is estimated that pollinators play a direct role in supplying up to 40% of the essential nutrients in the human diet [2]. Insect pollination alone contributes to 9.5% of the total economic worth of agricultural products that are directly consumed by humans [3]. Notably, developed nations like Britain, Germany, and Japan are expected to face the most significant economic repercussions due to the decline of pollinators [4].

Research conducted by Rodger, Bennett [5] has demonstrated that a reduction in pollinators can result in a 50% decrease in seed-based reproduction for approximately one-third of flowering plants, indicating that a substantial number of flowering plants rely entirely on pollinators for seed production. Consequently, plants that depend on pollinators are susceptible to shifts in plant–pollinator interactions. In Brazil, where pollinator-dependent crops constituted 47% of all dietary nutrients in 2017, a decline in pollinator populations could lead to nutritional losses ranging from 8% to 30% [5].

Temperature changes have direct and indirect effects on insects. Direct effects encompass the influence on the activity levels of larvae and adults, their geographical distribution, phenology (timing of life cycle events), and growth duration. Indirect effects involve alterations in host plant phenology, variations in food quality, and shifts in predator and parasite dynamics [6]. Over the past century, there has been an average global temperature increase of approximately 0.6 degrees [7]. Projections indicate that global warming could range from 1.5 to 5.8 degrees by the close of the 21st century [8].

It is well established that climate change and land use alterations have substantial implications for plant–pollinator relationships [9]. One of the most significant effects is the emergence of temporal and spatial mismatches [10,11]. Numerous studies have investigated the impact of climate change on insect pollinators, with a particular focus on bees. Many of these studies have examined limited insect species and geographic regions. In general, rising temperatures are likely to compel bee species to migrate towards higher latitudes and polar areas [12,13]. For instance, Rasmont, Franzén [14] predicted a decline in bumblebee diversity in Europe by 2050 due to climate change, suggesting that only specific regions, including mountainous areas, might maintain a significant portion of their existing diversity by 2100. Similarly, Suzuki-Ohno, Yokoyama [15] observed a reduction in the distribution range of five bumblebee species in Japan over 26 years attributed to climate change.

To anticipate the community-level consequences of climate change, it is essential to initially comprehend the individual responses of each species to these climatic shifts [16]. Recent studies have underscored that the impacts of climate change on certain pollinating insects have become increasingly evident in recent years. Projections for the future suggest that the distribution of these species will undergo alterations due to ongoing climate change. In addition, temperature fluctuations significantly influence the metabolic rates of insects. These variations play a pivotal role, often resulting in observable shifts in how these creatures regulate their energy and biological processes [17].

Pollinating insects encompass various groups such as moths, butterflies, bumblebees, honeybees, solitary bees, and hoverflies. Among these, bees hold particular importance as they are accountable for pollinating approximately 35% of the world’s food production [18]. Bees, categorized as ectothermic creatures, exhibit a significant reliance on the temperature of their habitat for their functioning. Honeybees and bumblebees are known to visit over 90% of global food crops [19]. However, to date, there has been a notable absence of research investigating the ramifications of climate change on the potential future ranges of bees on a global scale. Consequently, in this study, we aim to evaluate the potential impact of climate change on these species. To delve into the repercussions of climate change on the distribution of these essential pollinators, it is imperative to first establish a baseline understanding of their current geographic ranges. Subsequently, we will assess how each species might respond to anticipated shifts in climate conditions. Therefore, this study raises two pivotal questions: 1. What are the potential consequences of future climate changes on the global distribution of bees? 2. Which regions around the world are most susceptible to the effects of climate change on these species? By addressing these inquiries, we aim to shed light on the global-scale dynamics of pollinator distribution in a changing climate.

## 2. Materials and Methods

### 2.1. Occurrence Data

To acquire reliable and valid occurrence data for bees worldwide, we used the “*BeeBDC*” R package [20]. The “*BeeBDC*” is a novel R package and an extensive global bee occurrence dataset aimed at providing reliable species occurrence data for bees. By consolidating over 18.3 million bee occurrence records sourced from various public repositories such as GBIF, SCAN, iDigBio, USGS, and ALA, along with smaller datasets, Dorey, Fischer, Chesshire, Nava-Bolaños, O’Reilly, Bossert, Collins, Lichtenberg, Tucker and Smith-Pardo [20] proceeded to standardize the data, identify and remove duplicates, and refine the dataset using the reproducible “*BeeBDC*” R-workflow. This involved aligning species names based on established global taxonomy, unifying country names, and standardizing collection dates. Additionally, record-specific flags were incorporated to highlight potential data quality concerns. The dataset is made available in two formats: “cleaned” and “flagged-but-uncleaned” at https://doi.org/10.25451/flinders.21709757 (accessed on 10 October 2023).

### 2.2. Data Cleaning

In this study, we used the “cleaned” dataset comprising 6,890,148 bee records across six continents: Africa, Asia, Australia, Europe, North America, and South America. We took measures to ensure data integrity, starting with the removal of duplicate entries within the dataset. Subsequently, we isolated data for individual species based on the information provided in the “scientific name” column, resulting in the extraction of 11,607 species with at least one recorded occurrence. Our next step involved segmenting the records according to each continent and implementing a spatial filtering approach using the “Humboldt” R package [21]. This technique entailed setting a minimum distance threshold of 35 km between occurrence points, thereby mitigating spatial biases in the dataset. This methodology shares similarities with the spatial filtering methods employed in the “ENMTML” R package [22] under the “CELLSIZE” approach and the “FLEXSDM” R package [23] for occurrence data filtration purposes.

The number of occurrence data points necessary for creating a species distribution model (SDM) is influenced by several factors, including model complexity, data quality, species traits, research objectives, and scale [13,24]. While there is no fixed rule, some general guidelines apply. Generally, having a larger sample size with more occurrence data points is preferable because it provides richer information for modeling. A common rough guideline suggests aiming for a minimum of 30 to 50 presence points, but this requirement can vary significantly [24,25]. In our study, we set the minimum requirement at 50 data points per continent to enhance model precision. It is essential to note that the field of species distribution models (SDMs) has seen a wide range of studies with varying quantities of occurrence data, from as few as 10 [26] to several thousand data points [27].

Subsequently, we proceeded to filter out species with occurrence records of fewer than 50 from each respective continent. Consequently, we narrowed down the dataset to encompass 1365 bee species. A breakdown of the bee count per continent is detailed in Table 1. Furthermore, as part of our data verification process, we utilized ArcGIS software to clip the occurrence data, restricting them to the boundaries defined by the polygons representing the continents. Any data points recorded beyond these defined continental borders were omitted from the dataset to ensure data accuracy and reliability.

### 2.3. Environmental Variables

The physiology of ectothermic insects that are sensitive to climate plays a crucial role in their survival, development, and behavior. Ectothermic insects are cold-blooded, meaning they rely on external environmental temperatures to regulate their body temperature and metabolic processes. As a result, their physiology is intricately linked to climate conditions, and even minor fluctuations can have significant effects on their biology [28].

In this study, bioclimatic layers as predictor variables also were downloaded from the WorldClim database (www.worldclim.org, accessed on 20 October 2023). The bioclimatic data in the WorldClim database include 11 temperature variables and 8 precipitation variables. These 19 variables often have a high correlation with each other, and therefore, it is not recommended to use all these variables in species distribution modeling. For this purpose, we used the usdm [29] package to exclude the highly correlated variables from the set through a stepwise procedure based on the variance inflation factor (VIF) for each continent. 

The remaining variables include Mean Diurnal Range (Bio 2), Isothermality (Bio 3), Temperature Seasonality (Bio 4), Mean Temperature of Wettest Quarter (Bio 8), Precipitation of Wettest Month (Bio 13), Precipitation of Driest Month (Bio 14), Precipitation Seasonality (Bio 15), Precipitation of Warmest Quarter (Bio 18), and Precipitation of Coldest Quarter (Bio 19).

### 2.4. Model Fitting

Species distribution modeling is used to identify areas of high habitat suitability or species richness. Conservationists can use these models to prioritize areas for conservation efforts, such as establishing protected areas or implementing habitat restoration projects. With climate change affecting ecosystems and species distributions, SDMs can be used to assess how climate-driven shifts may impact species’ ranges [13]. To estimate the potential effects of climate change on pollinators, we used species distribution models (SDMs). 

In the present study, we used the “*FLEXSDM*” R package [23] to model the distribution of the studied species. For this purpose, we used the MaxEnt model to prepare climate suitability maps based on the presence and climate data. The MaxEnt model has been used in many studies to model the distribution of species and estimate climate change effects, especially on insects [30,31,32,33,34,35]. We also generated 5000 random pseudo-absence points to be used together with the presence points for modeling with the MaxEnt model in each continent. Next, we used the climate change scenarios of SSP585 (the shared socio-economic pathway) to model the future distribution of bees in 2070. The SSP585 is known as the worst scenario of climate change, in which CO_2_ emissions triple by 2075 and temperature increases by 4.4 °C by 2070 [36].

In the present study, we classified the climate suitability map into two classes of suitability, low and high, and the high-suitability class included values greater than 0.7 [13,37]. Therefore, we only measured range changes for the high-suitability class. Subsequently, we computed the percentage of change using the following formula: Percentage of change = ((number of cells in the future under the suitable class) − (number of cells in the current under the suitable class))/(number of cells in the current under the suitable class)) × 100.

### 2.5. Model Assessment

To evaluate the performance of the results obtained from different models, we used three statistics, namely inverse mean absolute error (IMAE), the area under the ROC curve (AUC), and Boyce Statistic (BOYCE), using the “*FLEXSDM*” R package [23]. The range of AUC values between 0.7 and 0.9 is considered acceptable, and values above 0.9 are considered excellent results, which means that the model has estimated a very good prediction. IMAE is calculated as 1 − (mean absolute error) to be consistent with the other metrics where the higher the value of a given performance metric, the greater the model’s accuracy. The Boyce Index serves as a measure for evaluating the predictive performance of species distribution models (SDMs) by assessing the agreement between observed and predicted probabilities across various levels of predicted suitability. Its values fall within the range of −1 to 1. When the values approach 1, they signify a strong alignment between observed and predicted probabilities, indicating the model’s effectiveness across diverse probability thresholds. Conversely, values hovering around 0 suggest the model’s performance is no better than random chance, indicating a lack of predictive power. Values less than 0 denote a performance worse than random chance, indicating the model’s poor performance in comparison to random predictions. We used 5-fold cross-validation [38] to evaluate the model’s performance. 

## 3. Results

### 3.1. Data Summary

After eliminating duplicate entries according to their geographical coordinates, the dataset retained 1,279,629 unique records. A summary of these records is displayed in Table 2, where they are categorized based on the proportionate representation within each family. Apidae emerges as the dominant family, contributing significantly with 1,024,488 records, encompassing an overwhelming 80.06% of the dataset. Halictidae follows, representing 82,423 records, equivalent to 6.44%. Further families include Andrenidae with 67,221 records, accounting for 5.25%; Megachilidae with 69,143 records at 5.40%; Colletidae registering 29,128 records, making up 2.28%; Melittidae contributing 7068 records with a proportion of 0.55%; and Stenotritidae appearing with 158 records, comprising merely 0.01% of the dataset.

### 3.2. Model Assessment

Table 3 shows the results of model validation metrics for different families based on statistics of AUC, BOYCE, and IMAE. According to this table, the value of AUC for all families is between 0.85 and 0.97, which indicates that the results of the models are acceptable. According to the other statistics, our predictions also fall within the perfectly acceptable range. The assessment outcomes regarding the performance evaluation for each species across individual continents are made available at https://github.com/ehsanrahimi666/ehsanrahimi666-Climate-Change-Effects-on-Bees, accessed on 20 October 2023.

### 3.3. Potential Effects of Climate Change

Table 4 outlines anticipated changes in the area of high suitability for species across continents by 2070 under the climate change scenario of SSP585. Africa’s projection lacks specifics on species increasing, yet it foresees a notable decrease in high-suitability areas for 44 species, declining by an average of 51.4%. In Asia, around 18 species might witness expanded high-suitability areas, increasing by an average of 99%, while 27 species might experience a reduction of 47%. Australia predicts increased suitability for 9 species (averaging a 16.3% rise) but signals a decrease for 97 species by 28.4%. Europe and North America both show a mix: Europe expects 60 species to gain in high-suitability areas (average increase of 57.9%) but anticipates decreases for 334 species, declining by 56.7%. Meanwhile, North America foresees gains for 366 species (an average increase of 48.2%) but predicts decreases for 331 species by 33%. South America’s projections exhibit potential for increased suitability for 19 species, with an average rise of 121.8%, yet 61 species might face reduced high-suitability areas, declining by 45%. These diverse forecasts across continents highlight the complex and varied impacts of climate change on species distribution, indicating both ups and downs in high-suitability areas of bees globally, thereby necessitating region-specific conservation strategies. The specifics regarding the proportional alterations of each species across individual continents can be found within the supplementary data (https://github.com/ehsanrahimi666/ehsanrahimi666-Climate-Change-Effects-on-Bees, accessed on 20 October 2023).

**Figure 1:**
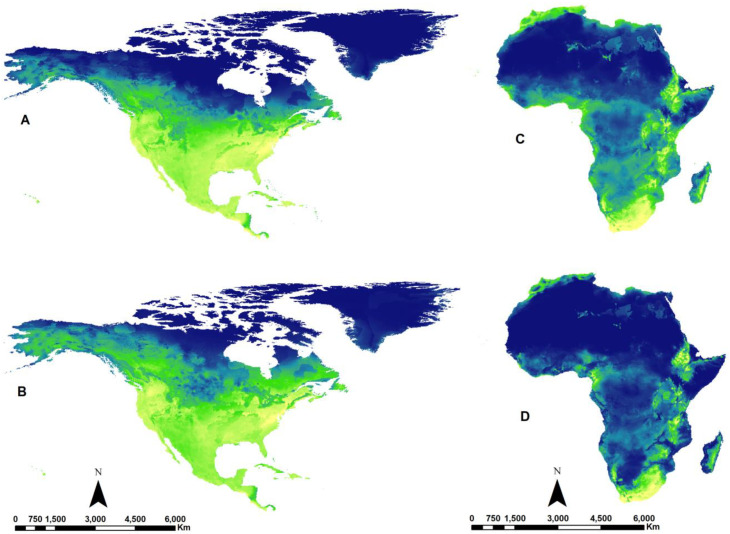
Climate suitability map of *Apis mellifera* (Hymenoptera: Apidae) in present (**A**,**C**) and future (2070) (**B**,**D**) in North America and Africa. Lighter colors indicate higher climate suitability, while darker colors represent lower suitability. The future climate suitability in North America might see an increase, but Africa may experience a decline. Refer to supplementary data for details (https://github.com/ehsanrahimi666/ehsanrahimi666-Climate-Change-Effects-on-Bees, accessed on 20 October 2023).

**Figure 2:**
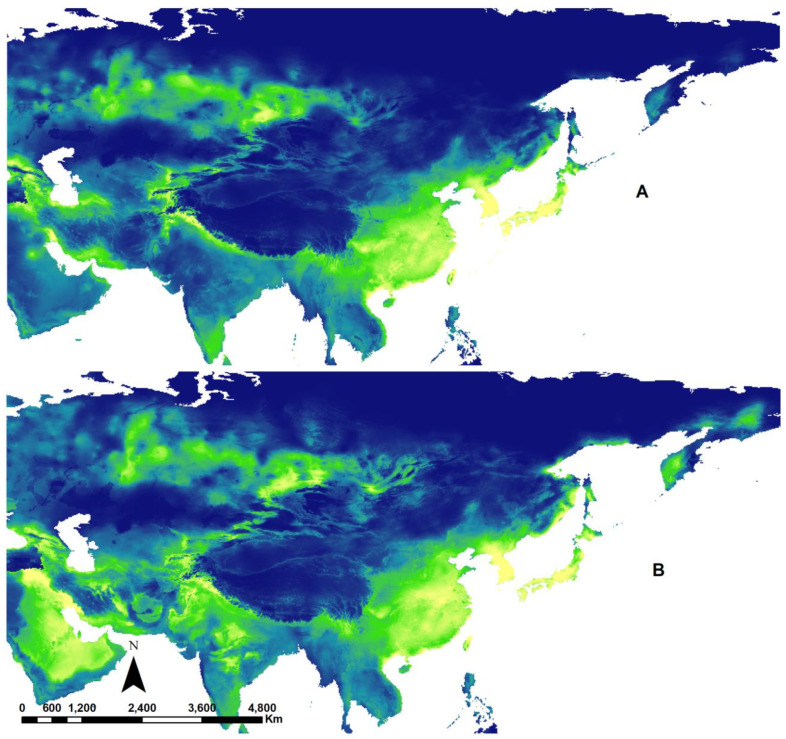
Climate suitability map of *Apis mellifera* (Hymenoptera: Apidae) in present (**A**) and future (2070) (**B**) in Asia. Lighter colors indicate higher climate suitability, while darker colors represent lower suitability. The changes in future climate suitability patterns are expected to increase in most parts of Asia. Refer to supplementary data for details (https://github.com/ehsanrahimi666/ehsanrahimi666-Climate-Change-Effects-on-Bees, accessed on 20 October 2023).

To gain a deeper understanding of the study’s findings and their implications for the impact of climate change on species by 2070, we selected *Apis mellifera*, as the most commonly observed floral visitor in natural environments globally [39] that serves as a primary pollinator for various crops worldwide [40], to illustrate how climate suitability could change due to the effects of global warming. Figure 1 illustrates climate suitability maps for *Apis mellifera*, both in the present and the future in North America (A and B) and Africa (C and D). In the current map for North America (A), the northern regions appear unsuitable, but in the future scenario (SSP585 in 2070), these areas become more suitable (B). Our findings suggest a potential increase in climate suitability for *Apis mellifera* in North America. On the right side, the map for Africa indicates a clear decrease in suitability for this species in the future (D). Figure 2 illustrates the climate suitability of *Apis mellifera* in Asia under both current (A) and future (B) climate scenarios. It visually demonstrates an expected increase in suitability in various parts of Asia, particularly in India, Saudi Arabia, Iran, and North Korea.

## 4. Discussion

We estimated the potential effects of climate change on 1365 bee species from seven families on a global scale. Our results for bee species exhibited varying responses to climate change in 2070. The extended time horizon utilized in our climate change scenario, spanning until 2070, poses inherent challenges due to the associated uncertainties and speculative nature of long-term projections. These uncertainties stem from various factors, including evolving climate models, potential socio-economic changes, and unforeseen technological advancements that may influence future climate trends. However, among bees, a majority, approximately 65%, are expected to see their range decline, with an average decrease ranging from 28% to 56%. Conversely, about 35% of bees are projected to encounter an increase in their distribution, averaging between 16% and 121%. Additionally, we found that Africa was the most vulnerable continent to climate change impacts on bee populations. Our critique raises an important point about the limitations of reporting bee range shifts on a global scale. Indeed, some bee species may experience extreme declines in certain parts of the world while increasing in others, and averaging these changes at the global scale may not accurately reflect the vulnerabilities of particular regions. Our research addressed this limitation by estimating the effects at the continental level, which can help to identify specific regions that are particularly vulnerable to climate change impacts on bees. This approach provided a more nuanced understanding of the potential effects of climate change on bees and can inform more targeted conservation efforts to protect these important pollinators.

Climate change scenarios typically involve rising temperatures. Most bees are ectothermic [41], meaning their body temperature is regulated by their environment. Warmer temperatures can potentially expand the suitable habitats for these species, allowing them to thrive in regions that were previously too cold. This expansion of temperature-appropriate zones can lead to an increase in distribution. Roughly 35% of the bee population examined in this study is anticipated to expand their geographic range, primarily favoring North America. However, this increase may not necessarily be advantageous for them because climate change can disrupt the timing of natural events, such as the flowering of plants and the emergence of bees [42,43,44]. Additionally, bees expanding their ranges may encounter new challenges, such as competition with native species or exposure to new predators and diseases [45,46]. Conversely, species facing distribution reductions may require targeted conservation strategies to mitigate the potential impacts on biodiversity and ecosystem services. Empirical findings based on fieldwork indicate that insects are already experiencing negative impacts from temperature changes, precipitation patterns, and other factors associated with climate change [47]. 

In the context of species distribution, it is typical to witness certain species expanding their ranges while others contract as a result of climate change. For example, Lima and Marchioro [48] anticipate that seven bee species will experience a decline in their suitable habitat, while three species will see an expansion in their suitable habitat in the future in Brazil. Dew, et al. [49] also found that as climate change continues, *Ceratina australensis* is expected to experience a broader expanse of suitable habitat. However, other research has reported a greater decline in the future distribution of bees attributed to climate change. For example, Giannini, et al. [50] investigated the effects of climate change on 216 bee species in the Eastern Amazon and found that 95% of the studied bees would experience a decline due to climate change. The observation that bumblebees have shifted to higher latitudes in recent years is also concerning, as it suggests that these insects are already experiencing significant impacts from climate change [12,15,51,52,53]. Indeed, it is important to acknowledge that the effects of climate change on bees may vary across species and regions. While some studies have shown that the distribution of certain bee species may decrease in response to climate change, other studies have found that some bee species may benefit from the changing climate and experience an increase in their distribution range [33,48,49]. These findings highlight the complexity of the issue and the need for careful consideration of individual species and regional differences in assessing the potential impacts of climate change on bees. 

Orr, Hughes, Chesters, Pickering, Zhu and Ascher [27] also employed the MaxEnt model to create a global species richness map for bees. Their findings revealed that bee hotspots are primarily concentrated in the southeastern United States, the Mediterranean basin, the Middle East, and Australia. Interestingly, tropical regions like Brazil do not display a high diversity of bee species. This distribution pattern aligns with the bimodal latitudinal pattern of bee richness described by [54], where warmer and drier areas appear to be more conducive to bee species diversity. Notably, xeric and non-forested regions play a pivotal role in driving bee biodiversity worldwide [27]. 

## 5. Conclusions

This study revealed the anticipated significant impact of climate change on global bee populations, indicating that a majority of species, roughly 65%, are likely to witness a decline in their distribution and suitable habitats, notably in Africa and Europe. Among continents, North America stands out with a considerable increase in bees expanding their distribution, contrasting with nearly 50% of bees projected to decrease in the future. While acknowledging the established role of non-bee pollinators, such as butterflies, moths, and hoverflies [55,56], in pollination, our research narrowed its focus to bees due to their recognized significance. Similar declines in distribution due to climate change are also being observed among other insect pollinators like butterflies, moths, and hoverflies [57,58,59,60,61]. It is important to note that in this study, our primary focus was on estimating the effects of climate change on bees. While our findings shed light on the impact of climate change, it is crucial to emphasize that habitat alteration, rather than climate change, is the primary driver of pollinator decline [62]. Habitat loss is commonly regarded as the primary driver behind the decline of bee populations [62,63,64]. 

## Figures and Tables

**Table 1:** Number of bees per continent with a minimum distance of 35 km and 50 occurrence records. Certain species exist across multiple continents.

Continent	Num. Species
Africa	44
Asia	45
Australia	106
Europe	394
North America	697
South America	79

**Table 2:** Summary of bee family distribution in dataset: counts and proportions.

Family	Count	Proportion%
Andrenidae	67,221	5.25
Apidae	1,024,488	80.06
Colletidae	29,128	2.28
Halictidae	82,423	6.44
Megachilidae	69,143	5.40
Melittidae	7068	0.55
Stenotritidae	158	0.01

**Table 3:** Model validation metrics including AUC, BOYCE, and IMAE generated using the MaxEnt algorithm for bees. The standard deviations are presented in parentheses.

Continent	AUC	BOYCE	IMAE
Africa	0.94 (0.03)	0.91 (0.05)	0.91 (0.04)
Asia	0.97 (0.02)	0.93 (0.03)	0.94 (0.04)
Australia	0.90 (0.06)	0.89 (0.04)	0.85 (0.01)
Europe	0.85 (0.04)	0.91 (0.03)	0.77 (0.07)
North America	0.95 (0.02)	0.90 (0.04)	0.92 (0.05)
South America	0.89 (0.08)	0.91 (0.04)	0.84 (0.11)

**Table 4:** The average percentage of changes in the area of the high-suitability class under the climate change scenario in 2070 in different parts of the world. The No. species column (increase/decrease) represents the number of species whose distribution range will change under the climate change scenario. The numbers in parentheses also show the standard deviations.

Continents	No. Species (Increase)	Increase%	No. Species (Decrease)	Decrease%
Africa	-	-	44	−51.4 (15)
Asia	18	99 (80)	27	−47 (19)
Australia	9	16.3 (18)	97	−28.4 (17)
Europe	60	57.9 (66)	334	−56.7 (21)
North America	366	48.2 (45)	331	−33 (22)
South America	19	121.8 (195)	60	−45 (23)

## Data Availability

The Excel data and the list of species presented in this study are openly available at https://github.com/ehsanrahimi666/ehsanrahimi666-Climate-Change-Effects-on-Bees, accessed on 20 October 2023.
